# Characterization of dorsal recumbency syndrome associated with woody breast in broiler flocks from Ontario, Canada

**DOI:** 10.1016/j.psj.2022.102307

**Published:** 2022-11-08

**Authors:** Sunoh Che, Lloyd Weber, Anastasia Novy, Shai Barbut, Leonardo Susta

**Affiliations:** ⁎ Department of Pathobiology, University of Guelph, Guelph, Ontario, N1G 2W1, Canada; † Department of Food Science, University of Guelph, Guelph, Ontario, N1G 2W1, Canada; ‡ Guelph Poultry Veterinary Services, Guelph, Ontario, N1L 1G3, Canada

**Keywords:** broiler chicken, meat quality, myopathy, serum chemistry, turtle bird

## Abstract

A dorsal recumbency syndrome (**DRS**) has been recently described in market-age broiler chickens. Affected broilers fall onto their backs, and are unable to right themselves, and eventually die of cardiopulmonary insufficiency. These broilers are referred to as *turtle chickens*. A previous report and anecdotal evidence suggest that breast myopathies, such as *woody breast* (**WB**), may be associated with DRS due to impaired contractility of the pectoral muscles. In this study, we aimed to provide additional evidence to document DRS in broilers, and its possible association with breast myopathies.

A total of 64 broilers (Ross 708), 33 DRS-affected and 31 controls, were culled between 42 and 48 d of age from 3 different commercial farms over 4 visits. All broilers underwent postmortem analysis; breast muscles were scored grossly and/or histologically to determine the presence and severity of myopathies, and sera were used to determine the level of aspartate aminotransferase (**AST**) and creatine kinase (**CK**).

A gross diagnosis of WB was moderately associated with DRS broilers, and DRS broilers displayed a greater microscopic severity of lesions (*P* < 0.001) in the *Pectoralis major*, as typically observed with WB. Levels of AST and CK were greater (*P* < 0.001) in the sera of DRS-affected compared to control broilers, consistent with muscular damage. The frequency of cardiac changes, such as mild hydropericardium and right ventricular dilation, or severity of microscopic pulmonary lesions, such as edema, were not significantly different between the 2 groups. The odds of DRS increased with the histology score of the *P. major* (OR = 1.37, 95% CI 1.02–1.85).

The data presented in this study support an association between DRS and muscular damage of the *P. major*, suggesting that WB may predispose broilers to DRS. DRS might be a cause of broiler death, and this syndrome could be responsible for significant financial loss to the farmers and to the whole poultry industry.

## INTRODUCTION

Dorsal recumbency syndrome (**DRS**) has been anecdotally reported by commercial broiler farmers in Ontario, Canada since 2018 (Shai Barbut, personal communication), and it has been documented in a single case report from the USA (Gall et al., 2019c). Broilers affected by DRS fall onto their backs due to an unknown cause, and later are unable to right themselves, a stance that has warranted the descriptive term “turtle chicken” used by some. Recumbent broilers eventually die of pulmonary failure of congestion and edema, possibly as a consequence of the pressure exerted on the cardiopulmonary system by the breast plate (Gall et al., 2019a,b,c).  While a recumbent dead broiler is also the typical presentation of sudden death syndrome (**SDS**, heart attack), it should be noted that SDS-affected broilers acquire a recumbent position due to flapping and convulsing in the context of acute heart failure (Newberry et al., 1987; Crespo, 2020). Instead, in DRS, broilers remain viable for a certain amount of time, as indicated by the fact that they can resume ambulating if helped to stand up again (Gall et al., 2019c). In fact, most broiler producers in Ontario cull DRS broilers, as these birds fall repeatedly even though caretakers place these broilers on their legs. The chronically affected broilers have wing trauma and ataxia which prevents broilers' access to feed and water (Lloyd Weber, personal communication).

It has been suggested that recumbent broilers are unable to right themselves because the *Pectoralis minor*, required to elevate (abduct) the wings, may not be able to exert a force on the humerus sufficient to antagonize the much more developed (i.e., hypertrophic) *Pectoralis major*, which lowers (adducts) the wings (Gall et al., 2019c). This impaired wing movement could contribute to keeping broilers lying with their backs on the floor.

As the improper function of the pectoral muscles has been suggested as a possible cause for DRS, breast myopathies, such as *woody breast* (**WB**), might also be associated with this condition. WB is a metabolic and degenerative disease that affects the breast muscles of fast-growing heavy broilers, and is characterized by heavy and firm pectoral muscles that histologically show myodegeneration, inflammation, and accumulation of connective tissue (Sihvo et al., 2017; Chen et al., 2019). It has been proposed that these lesions could cause a persistent contraction of the *P. major*, leading to defective abduction of the wing and the ultimate inability of recumbent broilers to right themselves (Gall et al., 2019c).

While WB is not a food safety issue, the textural changes within the breast fillets lead to a decreased consumers’ acceptance and cause the condemnation of severely affected breast fillets, leading to economic losses estimated to be approximately $1bn USD/yr (Barbut, 2020). Although WB has been regarded mainly as an issue for the poultry processors, little information is available regarding the possible negative impact of this condition on the health of broilers (Norring et al., 2019), especially considering its high prevalence in some flocks (Sihvo et al., 2017; Malila et al., 2018; Livingston et al., 2019; Che et al., 2022b). The economic burden of DRS has not been estimated yet, however, if breast myopathies were to be conclusively associated with DRS, their economic impact would reach beyond processing losses and meat quality.

In this study, we aim to provide additional characterization of the poorly documented DRS, and investigate a possible association between WB and DRS. We hypothesized that the gross and histological characteristics of pectoral muscles from DRS-affected broilers would be similar to those from WB-affected broilers.

## MATERIALS AND METHODS

### Inclusion Criteria

The cohort of broilers used in this study were collected from 4 flocks in 3 farms situated in Southwestern Ontario, Canada, between March 2020 and April 2021 (Table 1). Broilers were sampled as part of routine health checks and culling by the farm veterinarians. Broiler were classified as having DRS if they were found on their backs and were unable to right themselves but were alive (bright and alert). Control broilers did not present these signs, and were opportunistically collected as part of the normal culling for the flock. Dead broilers on the farm were excluded from our study. All experimental procedures involving animals followed the University of Guelph Animal Care and Use Committee, and were conducted in accordance with the relevant regulations.

**Table 1:** Demographics and tests carried out for each of the 4 flocks included in the study.

Flock ID	FSA	Age (d)	Sex		Targe weight (Kg)	Number of necropsies	Number of histology (*Pectoralis major*)	Number of WB scoring	Number of histology (lung)	Number of sera (AST & CK)	Number of sera (full profile)
1	N0K	45	Male		NA	3 controls 5 DRS	3 controls 5 DRS	NA	3 controls 5 DRS	10 controls 10 DRS	2 controls 2 DRS
2	N1M	42	Mixed		2.8	8 controls 8 DRS	8 controls 8 DRS	NA	8 controls 8 DRS	8 controls 8 DRS	2 controls 2 DRS
3	N1M	48	Male		4.2	10 controls 10 DRS	10 controls 10 DRS	10 controls 10 DRS	10 controls 10 DRS	10 controls 10 DRS	NA
4	N0B	41	Male		NA	10 controls 10 DRS	10 controls 10 DRS	10 controls 10 DRS	10 controls 9 DRS^1^	10 controls 10 DRS	NA
Total						31 controls 33 DRS 64 total	31 controls 33 DRS 64 total	20 controls 20 DRS 40 total	31 controls 32 DRS 63 total	38 controls 38 DRS 76 total	4 controls 4 DRS 8 total

Abbreviations: AST, aspartate aminotransferase; CK, creatine kinase; DRS, dorsal recumbent syndrome; FSA, forward sortation area; WB, woody breast.

1 one sample was missing.

### Sample Collection on the Farm

A total of 33 DRS and 31 non-DRS (control) broilers were collected (total, 64; Table 1). Broilers were euthanized on farms by cervical dislocation, and before euthanasia, blood was collected (approximately 5 mL) from the medial metatarsal vein and was allowed to coagulate in vials without additives (BD vacutainer serum tube 6 mL, BD, Franklin Lakes, NJ) to obtain serum. Sera from an additional 5 DRS and 7 control broilers, which were not necropsied, were obtained during the first visit (Table 1).

### Postmortem Examination and Histological Evaluation

Culled broilers were later transported to the Ontario Veterinary College, and a full postmortem examination was conducted on all 64 broilers (33 DRS and 31 control) to identify any gross lesions. Hearts were examined and scored for the presence of hydropericardium and right ventricular distension on a binary scale (0 = absent, 1 = present). From all carcasses, the heart, the lung, and the left cranial *P. major* were sampled, fixed in 10% buffered formalin, and processed for routine histology.

Before postmortem evaluation, the weights of the carcasses from 40 broilers sampled during visits no. 3 and 4 (20 DRS and 20 controls) were measured using a digital scale (PM600, Mettler Toledo, Columbus, OH). From the same birds, the dissected *P. major* was also weighed and scored for macroscopic evidence of WB using a binary scale (0 = absent, 1 = present). A positive score was assigned to tissues with severe WB changes, as previously described (Che et al., 2022b).

Histological scoring of *P. major* and the lung was carried out under a light microscope (BX45, Olympus Canada, Richmond Hill, ON, CA). Samples from the cranial area of the *P. major* were scored according to previously established criteria (Che et al., 2022a). Briefly, 24 areas measuring 1 mm^2^ each - identified using an adhesive grid - were scored for myodegeneration, perivascular inflammation, and endomysial accumulation of fat and fibrous tissue on a scale of 0 to 3. The histological score was calculated by adding the individual scores for each of the 24 squares.

Lungs were assessed microscopically, as it has been suggested that recumbent broilers might suffer from respiratory distress and show evidence of pulmonary edema (Gall et al., 2019c). Scoring of the lung considered edema and inflammation of parabronchi on a binary scale (0 = absent, 1 = present). The lung score was calculated by adding the scores of the affected parabronchi divided by the total number of bronchi per sample.

### Serum Chemistry

Serum samples were stored at – 80°C until sent to the Animal Health Laboratory, the American Association of Veterinary Laboratory Diagnosticians - accredited animal health laboratory for Ontario (Guelph, ON, Canada, https://www.uoguelph.ca/ahl/). Seventy-six samples were analyzed to detect the activity levels of aspartate aminotransferase (**AST**) and creatine kinase (**CK**), which are leakage enzymes released from damaged striated muscle (Kanda et al., 2014). Of these, 8 samples (4 DRS, 4 control broilers) were used for a complete avian biochemical panel, which included the following additional tests: the A:G ratio, albumin, amylase, bile acid, calcium, carbon dioxide, chloride, cholesterol, glutamyl transferase, glutamate dehydrogenase, globulin, glucose, hemolysis, lactate dehydrogenase, lipase, lipemia, phosphorus, potassium, sodium, total bilirubin, total protein, urea, and uric acid. All samples were analyzed using a biochemistry analyzer (Roche Cobas 6000 c 501, Roche Diagnostics, Indianapolis, IN).

### Statistical Analysis

#### Descriptive Statistics

Data were analyzed using a statistical software package (Stata 14.0; Stata Corporation, College station, TX). For biochemical analytes that were normally distributed, Student's *t* test was used for comparing differences between DRS and control broilers. The Wilcoxon rank-sum (Mann-Whitney) test was used for comparing the concentrations of AST, CK, and total bilirubin, as these were not normally distributed. The mean difference of histological scores in the *P. major* and lungs between DRS and control broilers was also tested using the Wilcoxon rank-sum (Mann-Whitney) test (Gibson-Corley et al., 2013). Statistical significance was set at 0.05.

#### Exploratory Statistics

Using samples from the third and fourth flock, Spearman's correlation test was performed to evaluate the correlation between presence and absence of DRS (binary), macroscopic presence of WB score (binary), carcass live weight (continuous), *P. major* weight (continuous), ratio of the *P. major* weight over live weight (continuous), hydropericardium (binary), right ventricle distension (binary), histology score of the *P. major* (continuous), histology score of the lung (continuous), AST concentration (continuous), and CK concentration (continuous).

Logistic regression analysis, implemented using Firth's penalized likelihood method (Firth, 1993), was conducted on the same cohort to estimate the correlation between the presence and absence of DRS (outcome variable) and the variables described above (explanatory variables). For logistic regression, AST and CK concentrations were dichotomized based on cut-off values. Specifically, AST and CK concentrations lower than 400 and 30,900, respectively, were considered normal and coded as 0 based on published reference values (Rodrigues et al., 2017; Livingston et al., 2020), whereas greater concentrations were coded as 1. Univariable analysis was initially performed between the outcome variable and independent variables using a relaxed *P* value (*P* = 0.2). Unconditionally significant variables were tested for collinearity by pairwise comparison using Spearman's rank test (Aggarwal and Ranganathan, 2016). Of the variables with strong correlation (rho > 0.70), only those with the lowest p value were retained. Remaining variables were included to a multivariable model.

## RESULTS

### Clinical Findings

**Figure 1:**
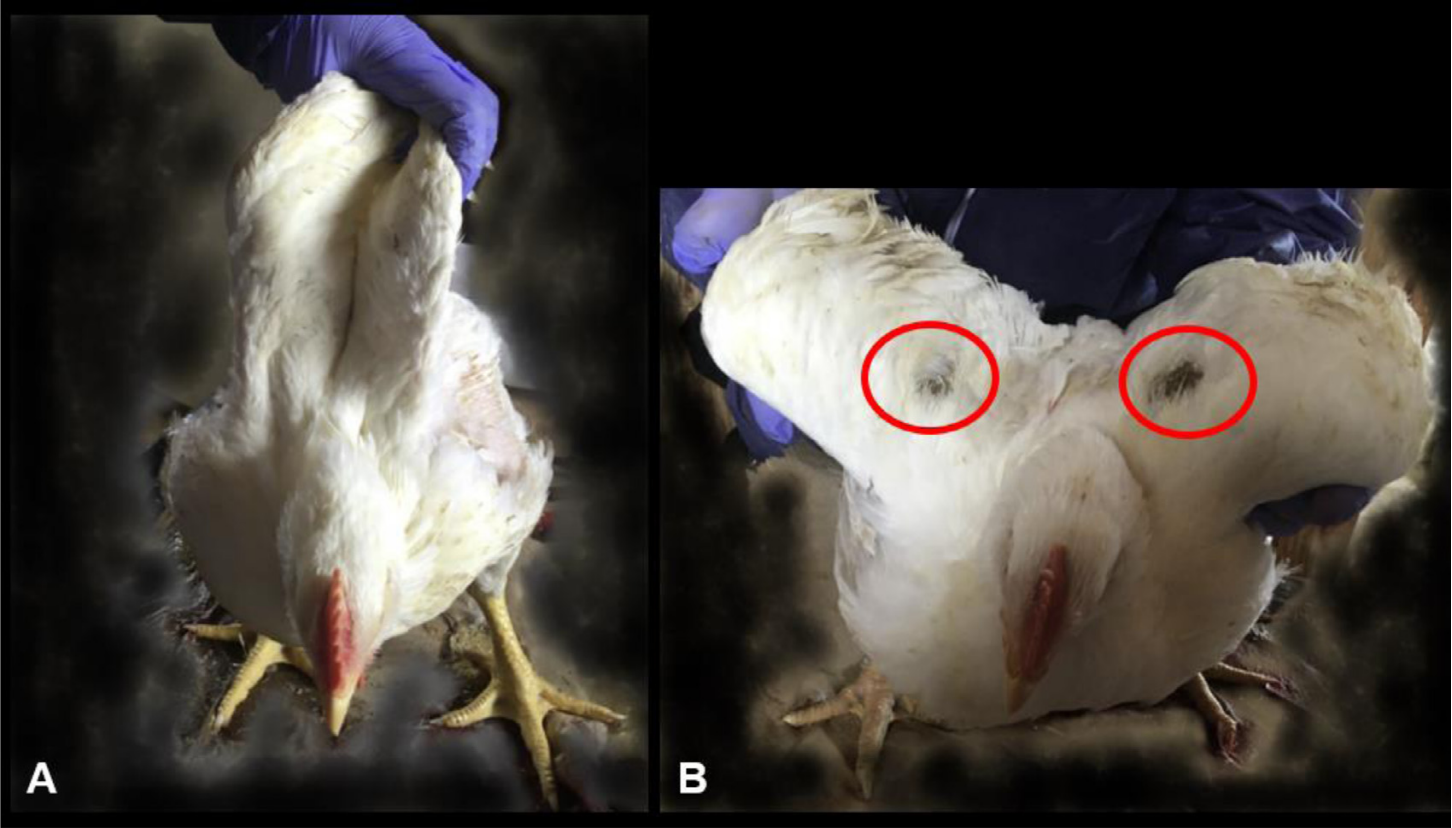
Representative photos from flock no. 4. (A) showing a control broiler that can lift its wings sufficiently to achieve back-to-back wing contact. (B) A broiler classified as recumbent chicken that cannot fully lift its wings and shows feather loss matting at the level the elbow joint.

Sixty-four broilers, 33 DRS and 31 control, were sampled from 4 flocks raised in 3 farms (Table 1). Flocks ranged from 41 to 48 d of age, 3 were male, and one was of mixed sex; market weight ranged from 2.8 to 4.2 kg. During observation, the DRS broilers were bright and alert and could move their legs. Once helped to stand, birds could resume walking although most of them could not fully abduct their wings, which could be lifted to only < 45^o^ angle (Figure 1). Some of the DRS broilers had skin ulcerations or feather loss at pressure points on their wings; most lesions were bilateral. For broilers sampled in flocks 3 and 4, 45% of DRS broilers showed bilateral matting of the feathers and small areas of ulceration at the level of the elbow (Figure 1). Decreased angular wing movement or dermatitis were not observed in control broilers.

### Postmortem Examination

In broilers collected from flocks 3 and 4 (20 DRS and 20 control), the body weight was measured, and the right *P. major* was dissected, assessed for macroscopic evidence of WB, and weighed. The DRS broilers (n = 20) weighed on average 3.46 kg ± 0.33; control broilers (n = 20) averaged 3.39 kg ± 0.27. The average *P. major* weight of DRS and control broilers was 358.6 g ± 77.4 and 355.5 g ± 74.7, respectively. The ratio of *P. major* weight over the carcass weight was 10.5 ± 2.6 and 10.5 ± 2.2 respectively, for DRS and control broilers. A total of 19 and 9 WB fillets were diagnosed in the DRS and control groups, respectively.

Necropsy indicated hydropericardium and distension of the right ventricle in both DRS and control broilers. Overall, 34.4% (95% CI 18.6–53.2) and 25% (95% CI 11.5–43.4) of DRS and control broilers, respectively, showed mild hydropericardium characterized by accumulation of < 1 mL clear and yellow fluid within the pericardial sac. Mild distension of the right ventricle was observed in 12.1% (95% CI 3.4–28.2) and 9.4% (95% CI 2.0–25.0) of DRS and control broilers, respectively. No additional macroscopic lesions were observed in the cohort of broilers used for this study.

### Histological Findings

**Figure 2:**
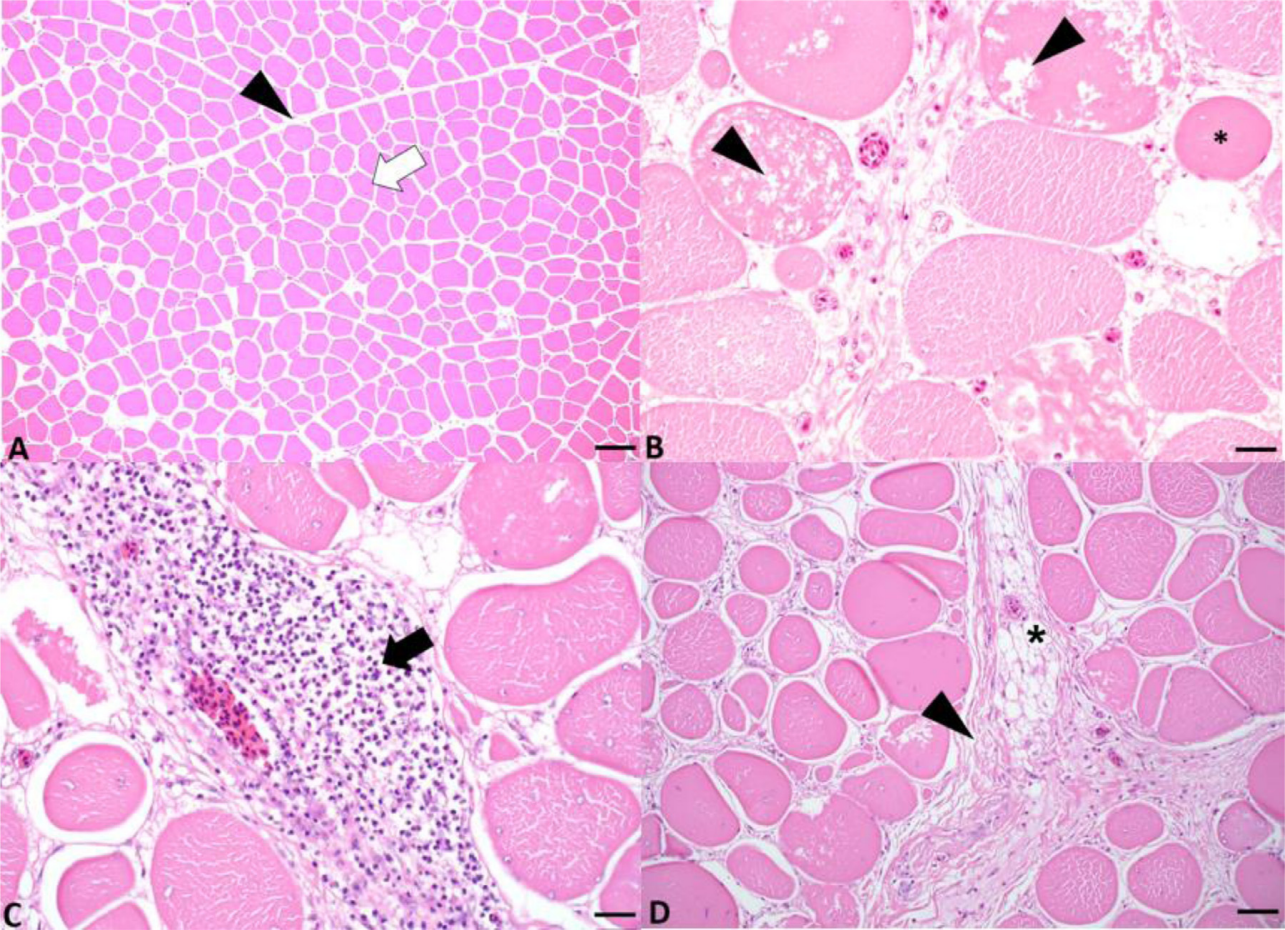
Histological images from *Pectoralis major* affected by dorsal recumbency syndrome and control broilers. (A) Polygonal normal myofiber (white arrow) and perimysium (black arrowhead); scale bar 100 um, in control bird. (B) Myodegeneration (black arrowhead), characterized by cytoplasmic vacuoles with floccular necrosis and irregular myofiber size, as shown by small round myofiber (*); scale bar 20 um, DRS broiler. (C) Perivascular infiltration of inflammatory cells (black arrow); scale bar 20 um, DRS bird. (D) Interstitial fibrosis (black arrowhead), lipid infiltration (*); scale bar 50 um, DRS bird. Hematoxylin and Eosin stain

The histological scores of the *P. major* from DRS-affected broilers were greater (*P* < 0.001) than those of control broilers (Table 2). *P. major* tissues affected by DRS showed myofiber degeneration and necrosis, which were characterized by loss of cross striation and fragmentation of the myofibers. Moreover, variations in muscle fiber size were observed, instead of the tightly packed polygonal myofibers observed in the control broilers. In the DRS broilers, the endomysium was multifocally expanded by fibrosis and adipocytes, and scattered intervening vessels were cuffed by numerous lymphocytes (Figure 2).

**Table 2:** Comparison of histological scores (mean ± SEM) of *Pectoralis major* and lung tissues between dorsal recumbent and control broilers.

Tissue	N	Category	Mean ± SEM	Min–max
*P. major*	10	Control broilers	10.8^a^ ± 1.5	2–25
*P. major*	10	Dorsal recumbent broilers	33.3^b^ ± 2.1	16–56
Lung	10	Control broilers	5.5^c^ ± 1.1	0–20.5
Lung	9	Dorsal recumbent broilers^1^	8.4^c^ ± 2.3	0–38.5

Abbreviation: N, number of samples.

1 Histology score of one dorsal recumbent broiler was missing.

a,b Different superscripts indicate means that are significantly different (Wilcoxon rank-sum test, *P* < 0.001).

Lungs in both groups showed rare parabronchi filled with homogeneous, lightly eosinophilic material (edema), or multifocal parabronchial inflammation, characterized by accumulation of rare heterophil, lymphocytes and fewer plasma cells. Only one broiler from the DRS group presented with a mild granulomatous pneumonia. There were no statistical differences in the histology scores of the lungs between DRS and control broilers (*P* = 0.820).

### Serum Chemistry

Serum concentrations of AST and CK were significantly higher (*P* < 0.001) in the DRS broilers compared to control broilers. The AST concentrations in DRS broilers was 1.6-fold greater than those of control broilers. Likewise, the CK concentrations in DRS broilers was 2.1-fold greater than those of control broilers (Table 3).

**Table 3:** Comparison of biochemical analytes (mean ± SEM) of dorsal recumbent broilers (n = 38) and control broilers (n = 38).

Biochemical analyte	Category	Mean ± SD	Min–max
AST (U/L)	Control broilers	501^a^ ± 261	218–1490
AST (U/L)	Dorsal recumbent broilers	852^b^ ± 435	263–2320
CK (U/L)	Control broilers	47700^c^ ± 42200	7630–161000
CK (U/L)	Dorsal recumbent broilers	99900^d^ ± 65900	3460–283000

Abbreviations: AST, aspartate aminotransferase; CK, creatine kinase.

a,b Different superscripts indicate means that are significantly different (Wilcoxon rank-sum test, *P* < 0.001). Numbers are rounded to 3 significant digits

Serum from DRS and control broilers did not reveal any differences (*P* > 0.05) in the amounts of analytes tested as part of the complete avian panel. Overall, serum chemistry data indicated that leakage enzyme associated with striated muscle had increased activity in the serum of DRS broilers compared to controls.

### Association Between Dorsal Recumbent Chicken and Postmortem, Histology, and Serum Chemistry Variables

**Figure 3:**
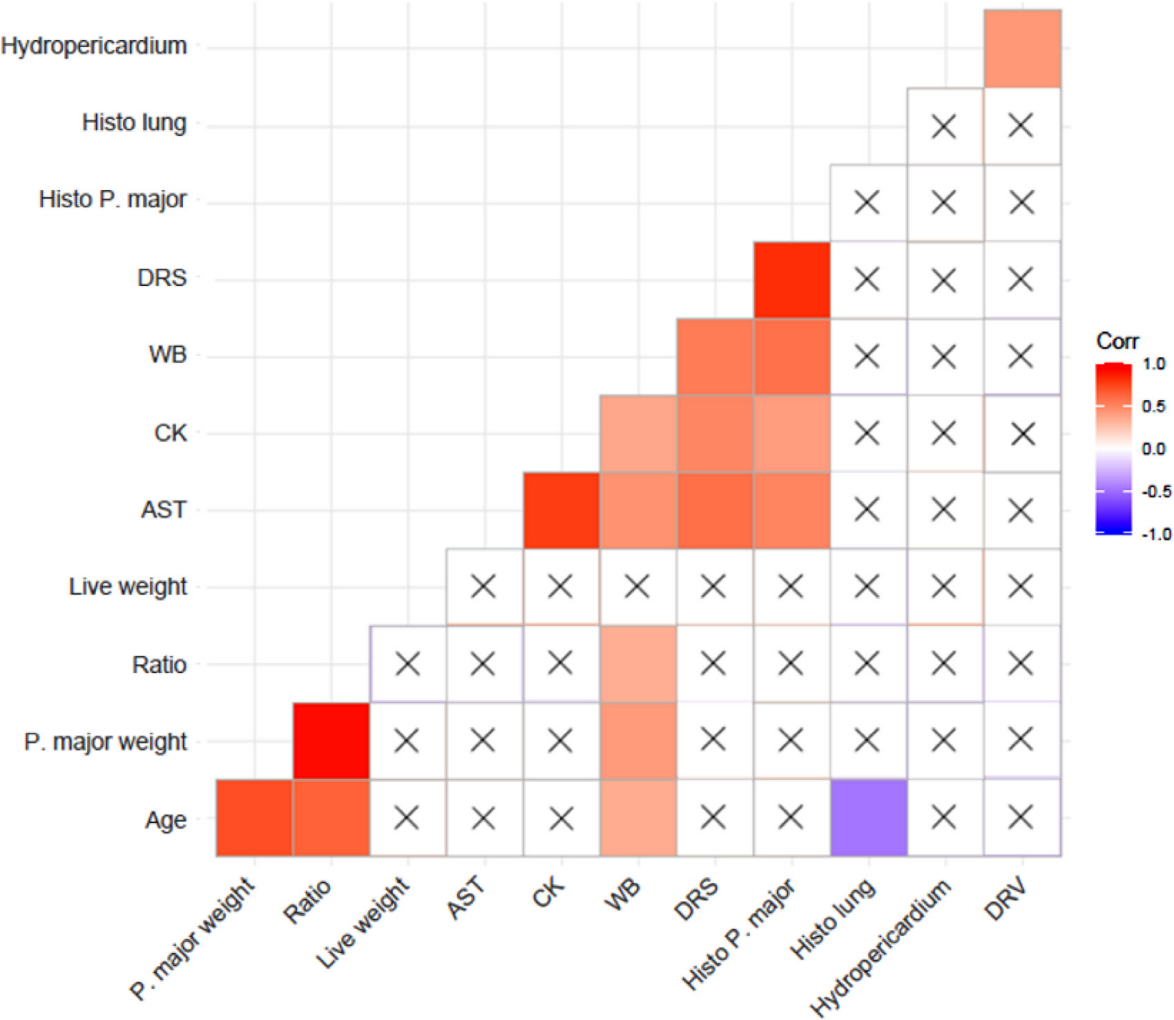
Correlation plot between diagnosis of dorsal recumbent syndrome (DRS), age, macroscopic diagnosis of woody breast (WB), live weight, *Pectoralis major* weight, ratio of the *P. major* weight over live weight (ratio), hydropericardium score, distension of right ventricle (DRV) score, lung histology score (histo lung), *P. major* histology score (histo *P. major*), aspartate aminotransferase (AST) concentration, creatine kinase (CK) concentration. In this analysis, only birds collected from flocks #3 and 4 were included (19 DRS and 20 control birds). Corr indicates Spearman's correlation coefficient: dark red shows a strong positive association, whereas dark blue shows a strong negative association. X indicates the correlation is not significant (*P* > 0.05).

The results of Spearman's correlation analysis are summarized in Figure 3. DRS and the histology scores of *P. major* were found to be strongly positively correlated (rho = 0.82, *P* < 0.001). There was a moderate significant association between the gross diagnosis of WB and DRS (rho = 0.54, *P* < 0.001). Moreover, DRS was moderately associated with AST concentration (rho = 0.59, *P* = 0.004) and CK concentration (rho = 0.50, *P* = 0.020), respectively. DRS was not associated with live weight, *P. major* weight, ratio of *P. major* and live weight, gross diagnosis of hydropericardium, distension of right ventricle, and histology score of the lung (*P* > 0.05).

The results of univariable logistic regression models are presented in Table 4. Out of 11 variables, 4 variables showed a significant association with DRS. The occurrence of DRS was associated with histology score of *P. major* (OR = 1.41, 95% CI 1.10–1.80), the macroscopic diagnosis of WB (OR = 22.00, 95% CI 2.44–198.14), high AST concentration (OR = 21.37, 95% CI 1.14–412.15), and high CK concentration (OR = 26.52, 95% CI 1.40–501.26). These 4 variables were not strongly correlated (rho < 0.60), and all were used for multivariable regression analysis. The final model (Table 5) shows that the occurrence of DRS was associated only with the histology score of *P. major* (OR = 1.37, 95% CI 1.02–1.85).

**Table 4:** Descriptive statistics and unconditional associations between 11 explanatory variables and the occurrence of the dorsal recumbent syndrome.

Variable	Number	Type	Value	Odds ratio	95% CI	*P* value
Age (d)	39	Continuous	41–48	1.01	0.85–1.21	0.869
Postmortem variables WB score	27	Categorical	Present	22.00	2.44–198.14	0.006
	12		Absent	Reference		
Live weight (g)	39	Continuous	2570–3925	1.00	0.99–1.00	0.403
*P. major* weight (g)	39	Continuous	252–555	1.00	0.99–1.01	0.710
Ratio^1^	39	Continuous	6.86–17.67	1.02	0.77–1.34	0.910
Hydropericardium	12	Categorical	Present	1.08	0.28–4.20	0.915
	27		Absent	Reference		
Mild distension of right ventricle	3	Categorical	Present	0.50	0.04–6.02	0.585
	36		Absent	Reference		
Histology variables Histology score of lungs	39	Continuous	0–38.5	1.04	0.96–1.13	0.339
Histology score of *P. major*	39	Continuous	2–56	1.41	1.10–1.80	0.006
Serum chemistry variables						
AST (U/L)	7	Categorical	Normal (218–312)	Reference		
	32		High (399–2318)	21.37	1.14–412.15	0.041
CK (U/L)	8	Categorical	Normal (7627–37072)	Reference		
	31		High (37178–137175)	26.52	1.40–501.26	0.029

Abbreviations: AST, aspartate aminotransferase; CK, creatine kinase; WB, woody breast.

1 Ratio of the *P. major* weight over liveweight.

**Table 5:** Results of multivariable logistic regression model to test correlation between diagnosis of dorsal recumbency syndrome and 4 explanatory variables.

Variable	Value	Odds ratio	95% CI	*P* value
Histology score of *P. major*	2–56	1.37	1.02–1.85	0.040
WB score	Present	1.27	0.06–8.43	0.880
	Absent	Reference		
AST concentration	Normal (218–312)	Reference		
	High (399–2318)	214.54	0.28–168680.2	0.115
CK concentration	Normal (7627–37072)	Reference		
	High (37178–137175)	0.01	0.0009–12.52	0.357

Abbreviations: AST, aspartate aminotransferase; CK, creatine kinase; WB, woody breast.

## DISCUSSION

The results of this study demonstrate that DRS-affected broilers are more likely to have damage of the *P. major.* Specifically, they are more likely to have lesions commonly reported with WB, which is a myopathy diagnosed with high prevalence in heavy broilers in most countries with intensive poultry rearing (Mutryn et al., 2015; Sihvo et al., 2017). Compared to control broilers, DRS broilers showed a significantly higher occurrence of WB, as diagnosed postmortem, as well as more severe microscopic lesions of the *P. major*. Microscopic lesions consisted of fibrosis, perivascular inflammation, and myocyte degeneration, which are common traits of WB, although not necessarily specific for this condition alone. Elevated serum levels of AST and CK were consistent with the observed muscular damage.

How damage of the *P. major* could induce dorsal recumbency is unclear. Wing movement requires coordinated actions of both the *P. major* (lowering the wings) and *P. minor* (elevating the wings), and it has been proposed that wing movement helps broilers re-establish an upright position once recumbent (Gall et al., 2019c). Since the *P. major* is in a state of permanent contracture due to WB (Sihvo et al., 2014; Velleman and Clark, 2015), contraction of the much smaller *P. minor* to elevate the wings is likely ineffective (Gall et al., 2019c). This ultimately causes the wings to remain flat against the ground, promoting a recumbent stance (Gall et al., 2019c). Decreased extensibility of the *P. major* could also account for the fact that WB-affected broilers are not able to sufficiently lift their wings to achieve back-to-back wing contact (Kawasaki et al., 2016). Similarly, the DRS-affected broilers in our cohort could not fully abduct their wings, supporting the hypothesis that increased contractility—or decreased extensibility—of the *P. major* plays a role in the development of DRS, and further suggests a relationship between WB and DRS.

Postmortem findings showed that DRS and control broilers in our cohort did not suffer from other concurrent severe diseases. Mild hydropericardium and mild distension of the right ventricle were observed without differences in frequency between the 2 groups, and were considered to be incidental findings. These changes are observed widely in heavy fast-growing broilers, and may be present subclinically (Julian et al., 1987; Olkowski, 2007; Olkowski et al., 2020). Similarly, lesions in the lungs were mild and considered incidental; and the severity of pulmonary lesions was not different between the 2 groups. In a previous study (Gall et al., 2019a,b,c), the authors reported that recumbent broilers presented with some degrees of respiratory distress and—if not eventually helped to right themselves—would die of cardiorespiratory collapse due to the weight of the internal organs and breast plate on the respiratory system (Julian, 1998). In the mentioned study, dead broilers presented with pulmonary edema and pericardial fluid accumulation, features that are also consistent with SDS (Ononiwu et al., 1979). SDS should be included in the list of differential diagnoses for broilers that are found dead on their backs, and it may not be possible to distinguish SDS and DRS broilers based on postmortem alone; thus, in our study only prospectively culled broilers were included. Therefore, we are confident that no SDS broilers were added to our study. Lack of spontaneously dead or terminally ill broilers could explain why we did not observe significant pulmonary or cardiac lesions, as these may develop only towards the latest stages of recumbency. Lastly, histology of the myocardium did not show vacuolation in the myofiber, fibrosis, and macrophage infiltration, which have been suggested as microscopic features of SDS (Olkowski et al. 2008).

It is not clear why broilers would fall in the first place. We did not observe angular deformities or tibial dyschondroplasia during our necropsies. Similarly, X-ray taken on carcasses of recumbent birds showed no bone abnormalities (Lloyd Weber, personal communication). There were no gross pathological changes in the brain and sciatic nerves during our postmortem examination. Thus, it is unlikely that DRS broilers had fallen due to neurological causes. It is possible that the heavy *P. major* muscle might have caused an imbalanced gait, promoting fall and recumbency (Norring et al., 2019). Alternatively, broilers might fall and become recumbent at low rates throughout the flock, with only predisposed broilers being unable to right themselves and becoming detectable. Interestingly, 45% of DRS broilers from flocks 3 and 4 presented matted and/or ulcerated pressure points on their wings. This suggests that some of these lesions might have been chronic, and might have been caused by more than one episode of recumbency. Wing lesions could also serve as supporting evidence to differentiate DRS and SDS.

Both CK and AST activity levels in the serum have been used as markers of skeletal muscle injury in broilers (Kuttappan et al., 2013; Ruiz-Jimenez et al., 2021). Our results show increased concentrations of AST and CK in the serum of DRS broilers compared to controls, and are in agreement with other studies that reported elevated levels of AST and CK in WB-affected broilers (Meloche et al., 2018; Kong et al., 2021). Increased activity levels of these enzymes are consistent with our histological findings, which show that pectoral muscles from DRS broilers were more severely affected by myodegeneration and inflammation, suggesting that AST and CK leaked out due to myocytes damage.

In our final multivariable regression model, 4 variables were retained and only one was significant. The model shows that the odds of diagnosing a recumbent broiler increase by 37% when the histology score of *P. major* increases by one point. We had previously shown that the histological grading scheme adopted in this study was positively associated with a gross diagnosis of WB (Che et al., 2022a). The other 3 variables (WB score, AST, and CK concentrations) were not significant in the final model, despite being associated with DRS diagnosis in the unconditional regression analysis. These variables may be confounders, being associated with the outcome and between each other (as different indicators of muscular damage), resulting in lack of significance in the model.

While WB has been associated with heavier carcasses (Griffin et al., 2018) and *P. major* muscles (Dalgaard et al., 2018; Bowker et al., 2019), diagnosis of DRS was not associated with these variables in our model. This might be due to the subjective scoring of WB (Petracci et al., 2019), the small sample size, or the fact that these weights may be poor proxies for WB, as it relates to DRS. There was also no association between hydropericardium or right ventricle distension and DRS. This is likely due to the fact that cardio-pulmonary lesions are detectable only in birds that died in a recumbent stance (Gall et al., 2019c), while in our cohort no terminally ill or dead birds were included (discussed above).

In conclusion, our study shows that the diagnosis of DRS is associated with gross and microscopic features of WB and serum indicators of muscular damage (AST and CK), with the histological score of the *P. major* being most stringently correlated with a diagnosis of DRS. As no comorbidities were observed in our cohort, our results suggest that WB may predispose broilers to DRS and negatively impact their health, as suspected by other investigators (Gall et al., 2019a,b,c) DRS might be a cause of broiler death prior to shipping to the processing plant, and this syndrome could be responsible for significant financial loss to the farmers and to the whole poultry industry. While this is a possibility, additional studies with larger number of birds are needed to establish causation and make sure that other predisposing factors might not have been overlooked. Moreover, additional studies to document the prevalence of DRS are needed to assess its impact on the health of commercial flocks, and establish a baseline to evaluate the effectiveness of mitigating measures.
